# Insights Into the Delivery of Personalized Nutrition: Evidence From Face-To-Face and Web-Based Dietary Interventions

**DOI:** 10.3389/fnut.2020.570531

**Published:** 2021-01-27

**Authors:** Balquees Al-Awadhi, Rosalind Fallaize, Rodrigo Zenun Franco, Faustina Hwang, Julie A. Lovegrove

**Affiliations:** ^1^Hugh Sinclair Unit of Human Nutrition and Institute for Cardiovascular and Metabolic Research, University of Reading, Reading, United Kingdom; ^2^School of Life and Medical Science, University of Hertfordshire, Hertfordshire, United Kingdom; ^3^Biomedical Engineering, School of Biological Sciences, University of Reading, Reading, United Kingdom

**Keywords:** face-to-face nutrition, personalized, personalized nutrition, web-based, dietary change

## Abstract

Prevention strategies for non-communicable diseases (NCDs) are a global priority as it has been estimated that NCDs will account for around 73% of worldwide mortality by the year 2020. The adoption of diets that are low in saturated fat, free sugars, and red and processed meats and higher in unsaturated fats, wholegrains, fruit, and vegetables have been shown to reduce the risk of NCDs. With increasing internet use, several nutrition interventions are now being conducted online as well as face-to-face, however it is unclear which delivery method is most effective. Although a consumer preference toward face-to-face dietary advice delivery has been identified previously, interest in delivering web-based dietary advice, and in particular personalized nutrition (PN), has been rising, as internet delivery may be less costly and more scalable. This review compares published face-to-face and web-based dietary interventions to give insight into which dietary method might be more effective for PN. In total, 19 peer-reviewed randomized controlled trials were identified for inclusion in the review. The majority of face-to-face nutrition interventions were successful at facilitating dietary change. Results from web-based nutrition interventions suggested that personalized web-based nutrition interventions may be successful at inducing short-term dietary change compared to standardized dietary interventions, however, minimal evidence of long-term impact has been found across both delivery methods. Results of a trial that compared face-to-face with web-based diet intervention found significantly greater dietary changes in the face-to-face group compared to web-based and control groups. Further controlled comparative studies and cost-benefit analysis are needed to assess whether web-based methods can be used in place of face-to-face interventions for achieving dietary change.

## Introduction

According to the World Health Organization, minimal physical activity (PA), obesity and poor dietary habits are major risk factors for non-communicable diseases (NCDs), which include cardiovascular diseases (CVD), type 2 diabetes and several cancers (1). In 2018, NCDs were responsible for around 89% of annual deaths in the UK and are the main cause of more than 2 million deaths annually in the European Union (1). Given that obesity is a major risk factor for NCDs, the adoption of a healthy lifestyle that includes a balanced diet and increased PA is essential to reduce the risk of NCDs (2).

Several studies have shown that the adoption of a diet that is relatively high in polyunsaturated fatty acids (PUFA), monounsaturated fatty acids (MUFA), potassium, fruit, vegetables; or moderately low in fat, saturated fatty acids (SFA), sodium, and dietary cholesterol may reduce the development of certain cancers and CVD (3–6). Despite public health campaigns, a significant proportion of the public is still not adopting this type of eating pattern (7), therefore additional counsel and intervention methods are necessary. Dietary advice can be delivered in several ways—via group or individual settings, over the phone, by text message, face-to-face with a dietitian/nutritionist (in person or via video call) or online and can therefore be given verbally and/or in written form. Face-to-face advice is typically provided by registered dietitians or nutritionists and involves tailoring or personalizing nutrition information to the individuals' requirements and lifestyle with the aim of facilitating behavior change.

Following technological advances, written methods of assessing dietary intakes and delivering dietary advice are being replaced or supplemented with computerized, web-based and mobile methods (8, 9). Based on findings from two systematic reviews, around 30 dietary trials have implemented remote methods to deliver the dietary information which included web-based, e-mails, videos, printed materials, and text messages (10, 11). Currently, most strategies used to either prevent or reduce obesity and CVD are based on standard public health recommendations and are therefore targeted at a population rather than individual level. For example, based on public overconsumption of salt and salt-rich products, public health messages aim to decrease consumption of salt as a protective method against stroke and other CVD (12). Nevertheless, more effective prevention strategies are necessary as NCDs continue to increase in number world-wide (2, 13).

Personalized nutrition (PN), that is, nutrition that is tailored toward an individual's or group's specific dietary requirements, has been identified as an important component of effective dietary intervention (14). PN may be more effective than general nutrition information as the advice is perceived as more personally relevant (15). One of the largest PN dietary intervention trial to date, Food4Me, used a web-based model to evaluate the efficacy of different levels of PN compared with standard population-based dietary advice, and found that PN improved dietary intake significantly more than non-personalized advice (16). Whilst Food4Me was delivered online, the research team identified a consumer preference toward face-to-face PN (17). However, face-to-face nutrition can be expensive, time consuming and may not be accessible to everyone (18). The use of web-responsive applications, websites, or emails provide an alternative method to face-to-face nutrition counseling that can reach a larger population. Web-based PN also allows individuals to access dietary interventions at home and therefore away from the usual clinical setting (19). Thus, interest in web-based health education messages has increased in recent years.

Given the differences in cost and reach between face-to-face and web-based nutrition, it will be useful to evaluate which method is more effective. Few trials have directly assessed the effectiveness of web-based nutrition intervention compared with face-to-face nutrition intervention. The purpose of this review is to assess evidence for the effectiveness of web-based and face-to-face dietary interventions on dietary change and to give insight into which method may be more effective at delivering PN.

## Methods

This review focuses on dietary change trials delivered in person/face-to-face or via the web in adult populations. A literature search was undertaken in PUBMED, Google Scholar, and MEDLINE to identify the effect of communicating dietary advice (to change dietary habits) in face-to-face and web-based settings. Terms used in the searches were face-to-face nutrition, nutrition interviews, weight-loss, dietary advice, web-based nutrition interventions, online, one-to-one nutrition counseling, online face-to-face nutrition, online one-to-one nutrition, Internet nutrition advice, obesity, dietary changes, and personalized nutrition. All terms were paired for outcome measures (dietary change). Only articles that were written or translated into English were included in the search.

### Study Selection

**Figure 1:**
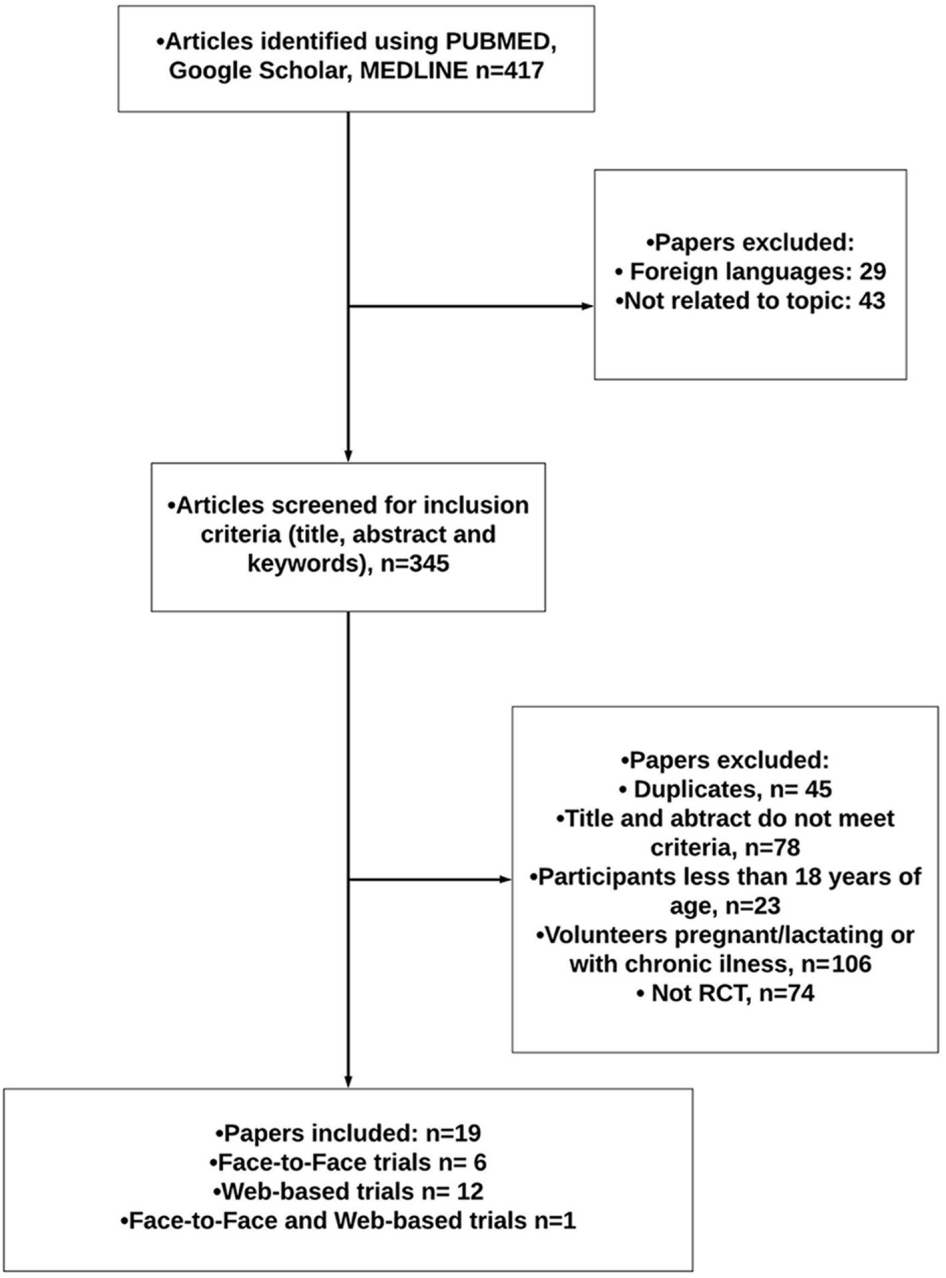
Literature search flowchart.

A total of 417 peer-reviewed and accepted manuscripts (from 1990 to 2020) reporting on RCT were identified; 19 were included in the review after screening (see Figure 1). Only randomized control trials (RCT) that reported original data on the effect of communicating dietary advice in a face-to-face setting or web-based nutrition interventions were included. Studies were excluded if the design trial was not a RCT or if the main focus of the trial was not dietary change. Face-to-face nutrition interventions included studies that utilized either individualized (one-to-one) settings or nutrition advice delivered in group settings. The focus of this paper was on dietary change for the healthy and overweight/obese population, therefore, studies were excluded if they were conducted with people with eating disorders, pregnant women and if the goal of the intervention was for treatment of a specific medical condition, with the exception of disorders with asymptomatic risk factors such as hypertension, hypercholesterolemia or impaired glucose tolerance.

## Results

Of the 19 RCT identified, 6 focused on face-to-face dietary interventions and 12 on web-based; 1 paper compared a face-to-face with a web-based dietary intervention. Furthermore, 16 out of the 19 articles incorporated PN as opposed to a standard dietary intervention.

### Face-To-Face Dietary Interventions

The face-to-face intervention studies identified (*n* = 6) were long-term trials (>6 months) that assessed the impact of dietary counseling on a variety of diet-related outcomes (e.g., specific nutrient intake, food groups/items). Four out of six of the face-to-face trials compared PN to generalized nutrition advice. Outcomes were either measured or self-reported (see Table 1).

**Table 1:** Face-to-face dietary interventions.

**Author**	**Number (*n*) and study characteristics**	**Baseline dietary data**	**Dietary assessment**	**Outcome(s)**	**Study findings**
Coates et al. (20)	*n* = 2,207 (I, *n* = 1324; C, *n* = 883) 18-month trial comparing the effectiveness of low-fat diets among post-menopausal women from several ethnic origins.	Fat (%): I group 39.74%, C group 39.08% F/V: 3.2 servings/d, C group 3.2 servings/d	FFQ	Fat (%): I group decreased by 13.3 vs. 2.3% in C group at 6 mo. and by −14.17 vs. −2.54% at 18 mo.; F/V: consumption increased by 0.5 serving/d in I group vs. 0.05 serving/d in C group at 6 mo. and by 0.8 serving/d and 0.1 serving/d in C group at 18 mo. Weight change: N/A	I group decreased percentage daily dietary fat intake and increased F&V intakes compared to baseline levels, but this change was non-significant. No change was seen for wholegrain foods.
Aldana et al. (21)	*n* = 348 (Diet, *n* = 174; C, *n* = 174) 6-month trial to determine the impact of a lifestyle-modification intervention receiving counseling compared to controls with no intervention.	Fat %: Diet group 36.7%, C group 34.6% F/V: 4.6 servings/d, C group 5.0 servings/d	FFQ	Fat (%): diet group lowered by 8.2% vs. increase of 1% in C group. F/V: diet group F increased by 0.9 serving/d vs. no change in C group and V increased by 1.4 serving/d vs. 0.1 in C group. Wholegrains: diet increased by 0.7 serving/d vs. decrease of 0.5 serving/d in C group. PA (steps/week): diet increased by 12,372 steps/week vs. 5,661 steps/week in C group. Weight change: Diet group −4.5 kg, C group −0.6 kg	At 6 months, diet group participants experienced significant improvements in all nutrition and PA variables except calories from protein and whole-grain servings (*p* <0.001).
Maskarinec et al. (22)	*n* = 29 (I, *n* = 13; C, n = 16) 6-month trial examining the effectiveness of increasing fruit and vegetable intakes among healthy women via personalized dietary sessions and group activities.	F/V: I group 3.2 servings/d, C group 3.3 servings/d	FFQ + 3 day DR	F/V: mean consumption increased in the I group by 5.1 serving/d at 3 mo. vs. 0.9 serving/d mean consumption in C group at 6 mo., F/V consumption in I group increased by 4.7 serving/d and C group increased by 0.8 servings/day compared to baseline. Weight change: N/A	Increased average F&V consumption in the I group at 3 and 6 months whereas minimal differences in intakes were found in the C group (*p* <0.001).
Steptoe et al. (23)	*n* = 271 (behavioral counseling; *n* = 13; basic counseling, *n* = 135) 12-month trial comparing brief nutrition counseling to behavioral dietary counseling	F/V: Behavioral counseling 3.7 servings/d, basic counseling 3.6 servings/d	FFQ	F/V: increased by 1.5 in behavioral counseling vs. 0.9 in basic counseling (5-a-day % increase) increased by 42% in behavioral counseling vs. 27% in basic counseling group. Weight change: N/A	Increased F&V intake in the behavioral counseling group compared to the basic counseling group at 12-months (*p* <0.021). % 5-a-day was also significantly higher in the behavioral group compared to the basic group (*p* <0.019).
Roderick et al. (24)	*n* = 956 (I, *n* = 473; C, *n* = 483) 12-month trial assessing the effectiveness of face-to-face dietary advice to generalized health information on serum cholesterol levels, diet, and weight	Serum Cholesterol: I group 6.0 mmol/, C group 6.2 mmol/l F/V: N/A Fat (%): I group 34.3%, C group 34.2%	FFQ	Serum cholesterol: I group decreased serum cholesterol by 0.20 mmol/l compared to C group 0.04 mmol/l. F/V: I group increased consumption of F by 0.76 serving/week and V by 0.33 serving/week vs. change of 0.28 F serving/week and −0.25 serving/week in C group. Fat (%): I group decreased by −2.4% vs. C group by −0.9%. Weight change: I group −0.1 vs. 0.44 in C group.	I group had lower mean serum cholesterol compared to C group. I group participants reduced their weight and intakes of dietary fat and saturated fat; this difference was not statistically significant.
Carpenter et al. (25)	*n* = 98 (Face-to-face group, *n* = 30; web-based, *n* = 33; C, *n* = 35) 14-week trial to assess the efficacy of group behavioral counseling via	Modified Healthy Eating Index: face-to-face group 61.2, C group 59.0 Fat score:	3 day DR	Modified Healthy Eating Index: face-to-face group increased fruit score by 2.2 vs. a reduction of 0.18 in web-based and 0.54 in C groups. Face-to-face group	Face-to-face group significantly improved scores compared to web-based (*p* = 0.04) and C group (*p* = 0.002).
	weekly meetings or correspondence to improve diet quality	Face-to-face group 4.9, C group 4.9		increased fat score by 2 vs. 0.81 in web-based group and 0.39 in C group. Weight change: N/A	
Baron et al. (26)	*n* = 368 (I, *n* = 187; C, *n* = 181) 12-month trial. examining the effectiveness of a dietary intervention that aimed at reducing blood lipid levels	Fiber: I group males 20.4 g/d & females 18.9 g/d, C group males 19.3 g/d & females 16.4 g/d SFA(%): I group males 67% used SFA & females 51%, C group males 47% used SFA & females 55% PUFA(%): I group males 20 and 19% females used PUFA, C group males 26 and 21% of females used PUFA	FFQ	Fiber (%): at 12 mo., I group reported to have increased daily % fiber by 52% male participants and 42% in female participants vs. 1% increase in males and 3% in reported fiber intakes in C group. SFA (%): I group males decreased SFA use % by 55% and females by 38 vs. 5% decrease in C group male participants and 0% fat change in females. PUFA (%): 22% increase in I group male participants and 30% in female vs. 1% in C group participants. Weight change: I group N/A or non-significant, C group –significant, C group nts.ts	I group reported increased intakes of fiber, PUFA and decreased use of saturated fat, minimal changes were reported in the C group. Differences between groups were statistically significant (*p* <0.001).

*N, total number of participants; I, intervention; C, control; F/V, fruits and vegetables; F, fruit; V, vegetables; PUFA, polyunsaturated fatty acids; FFQ, food frequency questionnaire; DR, diet recall*.

Aldana et al. Baron et al. and Maskarinec et al. all observed beneficial dietary changes following face-to-face intervention, when compared with no treatment controls. In the 6-month trial by Aldana et al. (*n* = 348) participants who received face-to-face lifestyle-modification intervention (40 h of diet and lifestyle group sessions with a registered dietitian) significantly improved F&V servings by 2.3 servings/d and decreased % daily intake of dietary fat by 8.2% with the exception of servings from whole grains and protein (% energy) when compared to baseline levels, and to control group participants (21). Baron et al. examined the effectiveness of a 12-month PN face-to-face vs. group nutrition intervention that aimed at reducing blood lipid levels vs. no treatment control. A total of 368 subjects were randomly allocated to either one of the two dietary intervention groups or a no treatment control group. The intervention groups were given dietary advice by a registered nurse either in a one-to-one or group setting. Following the trial, both intervention groups self-reported increased intakes of fiber by 47%, PUFA by 26%, and decreased intake of SFA by 40%; whereas minimal changes were reported in the no treatment control group and differences between groups were statistically significant (26). Results of the trial by Maskarinec et al. (*n* = 29 women) found that face-to-face personalized dietary counseling targeted towards F&V significantly increased dietary F&V intake (4.7 servings/d), when compared with general written dietary recommendations provided to controls (0.8 servings/d) (*P* < 0.001) (22).

Beneficial impacts of face-to-face interventions targeting dietary fat intake were reported by both Roderick et al. and Coates et al., however the results did not significantly differ compared with the control groups and to baseline values. Roderick et al. compared the impact of face-to-face PN intervention and generalized health information (usual care) on dietary intake. The 12-month study was targeted toward fat-loss (%), cholesterol reduction and changes in weight and a total of 956 participants were randomly assigned to either the intervention or a usual care control group. Following the trial, the intervention group had lower intakes of total dietary fat (2.4%) and lower mean serum cholesterol (0.20 mmol/l) compared to minimal changes in the control group, however these were not statistically significant between the groups and to baseline values (24). The 18-month trial by Coates et al. compared the effectiveness of low-fat diets in post-menopausal women (*n* = 208) that were randomly assigned to a low-fat intervention group or a control group that received paper-form Dietary Guidelines for Americans (27). In this study, participants in the intervention group were required to attend face-to-face group sessions with a nutritionist. At 6 months, the intervention group participants reduced their percentage of daily dietary fat intake (13.3%), but this change was non-significant from baseline. A non-significant increase in F&V intake was also observed in the intervention group (0.5 servings/d). Similar results were seen at 12 and 18 months of the trial (20).

Results of a 12-month trial that compared the impact of PN behavioral counseling to controls, that received brief nutrition counseling on improving F&V intakes, in 271 low-income adults found significantly increased F&V (1.5 servings/d) intakes in the individualized PN group compared to the control group. However, all F&V intake was based on self-report and participants received only two dietary consultations during the whole trial period (at baseline and week 2) that were restricted to 15 min (23).

Outcomes of the face-to-face dietary interventions suggest that in-person dietary advice, provided either individually or in group sessions, improved dietary intakes compared to controls. The majority of the trials have demonstrated significant improvements in number of F/V servings and dietary fat (%) intake in the face-to-face nutrition counseling groups compared to the control groups.

### Web-Based Dietary Interventions

Twelve PN web-based trials were identified (6 long term >6 months, 6 short term, 3–16 weeks) that assessed the effectiveness of web-based interventions at improving dietary change (Table 2).

**Table 2:** Web-based dietary interventions.

**Author**	**Number (*n*) and study characteristics**	**Baseline dietary data**	**Dietary assessment**	**Outcome(s)**	**Study findings**
Delichatsios et al. (28)	*n* = 298 (I, *n* = 148; C, *n* = 150) 6-month trial comparing the effectiveness a web-based dietary program that aimed at improving the overall health of individuals.	F intake (servings/d): I group 2.9, C group 2.7 V intake (servings/d): I group 4.1, C group 3.8 Fiber (g/d): I group 22, C group 21	FFQ	F/V: consumption of fruit increased in the I group by 1.1 serving/day compared to C group. No difference for vegetables. Fiber: increased by 4.0 g/d in I group compared to C group. Weight change: N/A	I group significantly increased their fruit from baseline levels compared to C group (*p* <0.05).
Anderson et al. (29)	*n* = 277 (I, *n* = 129; C, *n* = 148) 6-month trial comparing the impact of a web-based intervention on the food choices made by supermarket shoppers.	Fat (%): I group 33.2%, C group 32.7% F/V(servings/1,000 Kcal): I group 2.8, C group 2.8 Fiber (servings/1,000 Kcal): I group 8.9, C group 8.9	FFQ + Food shopping receipts	Fat (%): decreased by 9% in I group vs. increased by 2% in C group. F/V (%): consumption increased by 20% in I group vs. 2.8% decrease in C group. Fiber (%) 19% in I group vs. 4% decrease in C group. Weight change: N/A	I group decreased their fat intake by 9% (*p* <0.05) and increased their serving sizes from F&V by 20% and (*p* <0.01) increased their total fiber intake by 19% (*p* <0.001). C group increased their total fat intake and had slightly lower fiber intake.
Stevens et al. (30) Celis-Morales et al. (16)	*n* = 616 (I, *n* = 308; C, *n* = 308) 4-month trial to assess the efficacy of a web-based counselling intervention to minimise risk of diet-related cancers. *n* = 1,269 (PN diet, *n* = 312; PN diet + phenotype, *n* = 324; PN diet + phenotype + genotype, *n* = 321; C, *n* = 312) 6-month trial to examine the effectiveness of PN advice on dietary change in comparison to “one size fits all” advice.	Fat (%): I group 33.1, C group 32.2 F/V (Servings/d): I group 5.1, C group 5.0 Red and processed meat (g/d): PN I groups 79.2, C group 74.4 Salt (g/d): PN I groups 7.4, C group 7.3 HEI: PN I groups 49.1, C group 49.5	24 h DR FFQ	Fat (%): I group lowered fat by 2.84 vs. C group increased by 0.48. F/V: I group increased by 0.54 serving/d vs. lowered by 0.51 serving/d in C group. Weight change: N/A Red and processed meat intake decreased by 8.5%, salt intake decreased by 6.3%, energy intake decreased by 4.4% in all three PN intervention group compared to C group. HEI increased by 2.7% in PN intervention groups compared to C group. Weight change: PN I groups −2.8 kg, C group −0.5 kg	At 4-months, I group had significantly increased F/V servings/day (*p* <0.001) and decreased daily fat % intake significantly compared to C group (*p* <0.009). At 6-months, PN intervention groups improved intakes of red and processed meats, salt, had lower energy intakes and increased HEI significantly compared to C group (*p* <0.05).
Brug et al. (31)	*n* = 507 (I, n =178; C, *n* = 169) 6-week trial examining the effect of online personalised nutrition information on fat intake and fruit and vegetable intakes.	F/V (servings/d): I group 2.5, C group 2.6 Fat (fat points/d): I group 29.0, C group 28.0	FFQ	F/V: minimal increase in I group in F intake by 0.008 serving/d and V by 0.04 serving/d and C group decreased F intake by 0.04 serving/d and increased V by 0.06 serving/d. Fat: fat points/d decreased by 2.1 in I group compared to 0.8 in C group. Weight change: N/A	Minimal increase in F&V consumption was found in the I group from baseline levels. Fat intake decreased significantly in the I group (*p* <0.001) and C group (*p* <0.05) compared to baseline.
Block et al. (32)	*n* = 481 (I with phone calls, *n* = 162; I without phone calls, *n* = 160; C group, *n* = 159) 8-week trial to assess whether an interactive CD-ROM can enhance the diet of low-income women.	F/V (servings/d): N/A	Diet survey + 24 h DR	F/V: I with phone calls increased by 1.32 serving/d vs. I without phone calls by 1.20 serving/d vs. 0.71 serving/d in C group. Weight change: N/A	After 2 months, both intervention groups significantly increased F/V consumption compared to C group (*p* <0.016 I with phone calls group, *p* <0.052 I without Phone calls group).
Alexander et al. (33)	*n* = 2,540 (I1, *n* = 848; I2, *n* = 845; C, *n* = 847) 12-months trial to assess F&V intake by comparing online tailored to non-tailored dietary interventions.	F/V (servings/d): I1 3.3, I2 3.2, C group 3.4	FFQ	F/V: I1 increased by 2 servings/d vs. I2 increased by 2.8 servings/d vs. C increased by 2 servings/d. Weight change: N/A	Average F&V servings increased by more than 2 servings across all study arms (*p* <0.001). Greatest increase in I2 compared to C group at 12 months (*P* = 0.05).
Irvine et al. (34)	*n* = 517 (I, *n* = 260; C, *n* = 257) 2-month trial comparing the effectiveness of an interactive computer -based program on the dietary intake of individuals.	Fat (DHQ): I group 2.5, C group 3.0 F/V (DHQ): 3.0, C group 3.1	FFQ	Fat, F/V: I group decreased fat DHQ score by 0.5 SD and increased F&V intake by 0.93 SD compared to baseline levels vs. a 0.41 SD decrease in fat score	After 1 month, the I group reduced their fat intake compared to the C group (*p* <0.001). I group significantly increased F&V consumption
				and 0.88 increase in F&V intake in C group. Weight change: N/A	compared to controls (*p* <0.001). I group maintained these dietary changes after a 60 day-follow up.
Oenema et al. (35) Collins et al. (36)	*n* = 782 (I, *n* = 261; G, *n* = 260; C, *n* = 261) 3-week trial examining effectiveness of a short-term computer tailored nutrition intervention that aimed at decreasing saturated fat intakes and increasing fruit and vegetable intakes and to raise personal dietary awareness. *n* = 65 (I, *n* = 34; C, *n* = 31) 3-month trial examining the effectiveness of the SHED IT web-based intervention on weight loss and dietary change in overweight/obese men.	Fat (points): I group 19.8, G group 20.0, C group 20.3 F/V (servings/d): I group 4.0, G group 3.9, C group 4.0 PSF: I group 1.5, C group 1.5 Fat (%): I group 35%, C group 35% SFA (%):I group 15%, C group 15%	FFQ FFQ	Fat (points): I group decreased by 0.6 vs. 0.8 in G vs. 0.4 in C group. F/V: V intake Increased by 0.1 serving/d in I group vs. lowered by 0.1/serving/d in GI vs. lowered by 0.1 serving/d in C group. Weight change: N/A PSF: decreased in both I and C group to 1.3. Fat (%) I and C group reduced to 32%. SFA I and C group reduced to 13%. Weight change: I group −5.3 kg, C group −3.5 kg	I group significantly increased their awareness of the benefits of consuming a diet high in fruits and vegetables (*p* <0.05) and low in fat compared to G and C group. Both I group and C group significantly reduced daily energy intakes (*p* > 0.001), percentage energy from fat (*p* <0.05) and SFA (*p* <0.001).
Vandelanotte et al. (37)	*n* = 771 (simultaneous group 1, *n* = 189; sequential group 2, *n* = 180; sequential group 3, *n* = 204; C, *n* = 194) 6-month trial comparing the effectiveness of a computer-individualised intervention on dietary fat intake and physical activity	Fat (%): I1 40.8, I2 + I3 38.0, C group 35.3 PA (min/week): I1 532, I2+I3 514, C group 720	FFQ	Fat (%): I1 decreased by 11.5%, I2 + I3 groups by 8.6% compared to 2.1% in C group. PA: increased by 61 min/week in I1 and by 93 min/week in I2 + I3 and by 45 min/week in C group. Weight change: N/A	I1, I2, and I3 groups significantly increased their PA scores (*p* <0.001), and reduced fat intakes (*p* <0.001) when compared to C group participants.
Winett et al. (38)	*n* = 141 (I, *n* = 54; C, *n* = 51) 10-week trial examining the effectiveness of a computer-based program “The Nutrition for a Lifetime System” (NLS) that aimed at helping shoppers at supermarkets to decrease intakes of fat and increase intakes of fruits, vegetables and fiber	F/V (servings/d): I group 1.4 C group 1.4 Fat (%): I group 38.4, C group 38.7 Fiber (g/1,000 Kcal): I group 6.2, C group 6.9	Food shopping receipts	F/V, Fat, Fiber: F/V I group increased F/V intake by 0.29 serving/1,000 Kcal compared to −0.12 serving/1,000 Kcal in C group. Fat (%): I group decrease by 3.2% compared to 0.7% increase in C group. Fiber: I group increased by 1.24 g/1,000 Kcal compared to decrease of 0.61 g/1,000 Kcal in C group Weight change: N/A	I group significantly reduced their fat intake and increased their intakes of fiber and F&V compared to C group participants (*p* <0.001).

*N, total number of participants; I, intervention; C, control; I1, intervention 1; I2, intervention 2; I3, intervention 3; G, general nutrition information; F/V, fruits or vegetables; F, fruits; V, vegetables; SD, standard deviation; PA, physical activity; DHQ, diet habits questionnaire; FFQ, food frequency questionnaire; DR, diet recall; HEI, healthy eating index; N/A, not available; PSF, portion size factor*.

Two of the trials examined the effectiveness of the Nutrition for a Lifetime System (NLS) web-based program, with comparable results (29, 38). The NLS is an automated computerized intervention based in supermarkets and provided PN feedback on personal behavior change goals. The first 6-month trial by Anderson et al. compared the impact of the computer-based intervention on the food choices made by supermarket shoppers (29). Participants (*n* = 277) were randomly assigned to either a PN computer-based intervention group that received the NLS web-based program or a no-treatment control group. At 6-months, participants in the intervention group significantly decreased their fat (9%) intake from baseline and significantly increased intakes of F&V (20%) and fiber (19%) (29). Similar findings were achieved in a trial by Winett et al. that examined the effectiveness of the NLS. Participants (*n* = 127) were randomly assigned to either the PN NLS or a control group for 10-weeks and were asked to return supermarket weekly purchase receipts. Participants in the PN NLS intervention completed the NLS computer program weekly and control group participants did not receive dietary advice. Participants in the PN NLS group significantly reduced their fat (3.2%) intake and increased their intakes of fiber (1.2 g/1,000 kcal) and F&V (0.29 g/1,000 kcal) compared to control group participants (*P* < 0.001) (38).

Results of the EU Food4Me 6-month web-based dietary intervention study found that web-based PN, regardless of the level of personalization, was more effective at improving healthy eating, when compared to controls that received standardized web-based dietary advice (16). Participants (*n* = 1,269) were randomized to PN dietary advice, PN dietary advice + phenotype, PN dietary advice + phenotype + genotype or to a generalized dietary advice control group. At 6 months, participants in the PN groups had significantly lower intakes of red and processed meat (8.5%), salt (6.3%), daily energy intakes (4.4%), and significantly improved their overall Healthy Eating Index (HEI) (2.6%) scores compared to participants in the control group *P* < 0.05 (16).

Results of the 3 other long-term web-based dietary interventions also found that web-based PN interventions can result in significant dietary improvements when compared to a control group (28, 33, 37). Results of a 12-month trial on 2,540 volunteers that compared two PN web-based interventions (PN intervention, PN + motivational e-mail counseling intervention) to a non-personalized intervention control group, indicated that the PN intervention with motivational counseling resulted in significantly greater F&V intakes (2.8 servings/day) when compared to controls (2 servings/day) (*P* = 0.05). All groups increased F&V intakes significantly at the end of the trial compared to baseline values (33). A 6-month dietary intervention study by Delichatsios et al. examined the effectiveness of a web-based PN program that aimed at improving several aspects of diet quality. Adults (*n* = 298) were randomized to either the PN intervention group who received weekly sessions with a computer automated voice program or the control group that received web-based PA information. At 6 months, intervention group participants increased intakes of fruit (1.1 servings/d) and fiber (4.0 g/d) significantly compared to control group (*P* < 0.05) (28). A 6-month trial by Vandelanotte et al. that compared a PN web-based dietary change intervention to a wait-list control, produced similar results. Participants (*n* = 771) were randomly assigned to four groups; the first group received PA and fat intake information at baseline, second group received PA information at baseline and fat intake information at 3 months, group 3 received at baseline the fat intake information and PA information at 3 months or a group 4 wait-list control group. All PN intervention groups significantly increased their PA scores (77 min/week), and reduced fat intakes (10%) when compared to control group participants (37).

Results of the 6 short-term web-based dietary change trials found significant differences between intervention and control groups (30–32, 34–36). A 12-week trial by Brug et al. examined the effectiveness of a PN web-based intervention on total fat and F&V intakes. Participants (*n* = 347) were randomized to the PN intervention group, that received online feedback based on their dietary intakes, or a non-personalized control group that received generalized nutrition related information. Participants in the PN group significantly decreased their fat score by 9% compared to baseline levels and to the control group (*P* < 0.01). However, fruit consumption in the PN group remained similar to baseline (31). Findings from a 12-week SHED-IT intervention on 65 overweight/obese men that assessed dietary, PA and weight loss changes using PN web-based feedback reports were successful at reducing fat and saturated fatty acid intakes. Participants were randomized to a web-based group that received personalized feedback reports on specific dietary areas (sodium, fiber, saturated fatty acids, and calorie intake) or to a control group that were provided with a dietary and PA handbook. Trial results have shown significant improvements in both groups in portion size factor (PSF) (1.3), fat (32%), and SFA (13%) compared to baseline values, however, non-significant differences were found between the groups (*P* < 0.05) (36). A 12-week trial by Irvine et al. evaluated the effectiveness of a PN interactive behavioral change computer-based program on the dietary intake of individuals. Participants (*n* = 517) were randomized to either an intervention or control group. After 1 month, the intervention group significantly reduced their fat intake diet habit questionnaire (DHQ) score (2.27) and increased their DHQ score for F&V intakes (3.36) compared to controls (*P* < 0.001). Furthermore, the intervention group maintained these dietary changes after a 60-day follow up (34). An 8-week-long trial assessed the efficacy of web-based dietary change programs to improve the dietary intake of 481 low-income women. Subjects were allocated to a PN web-based group, PN web-based + phone-calls with researcher group or a non-diet related control group. Results of the trial indicated F&V intakes increased in both groups (1.3 servings/d) compared to controls, which reached borderline significance (*P* = 0.05) (32). Research by Oenema et al. studied the effectiveness of a short-term web-based PN intervention that aimed at improving dietary awareness. A total of 782 subjects were randomly assigned to a PN intervention group or a general nutrition control group or a control group that did not receive any information for a 3-week period. The intervention group significantly increased their awareness of the benefits of consuming a diet high in F&V (0.6 points) and low in fat (0.1 servings/d) compared to the control groups (35). Comparable results were found in a 4-month trial by Stevens et al. that examined the effectiveness of a web-based PN intervention to improve dietary intake compared to controls. A total of 616 women were randomized to either the intervention group that received access to a web-based PN program in addition to two counseling sessions or a control that received non-diet related information. At the end of the trial, the PN intervention group significantly increased F&V intake (0.54 servings/d) and decreased fat intake (2.8%) compared to controls (*P* < 0.001) (30).

Results of these trials suggested that web-based PN dietary advice was effective at enhancing dietary change compared to standardized controls. Outcomes of 10 trials indicated significant improvements in either F/V or % daily fat intake in the groups that received web-based PN dietary advice in comparison to control group participants.

### Face-To-Face Compared With Web-Based Dietary Intervention

A 6-month cognitive and behavioral pilot study by Carpenter et al. compared the effectiveness of face-to-face and web-based interventions on dietary change. A total of 98 volunteers were randomized to a face-to-face group, a web-based with personalized email feedback group or a control group. The face-to-face group met with a counselor once/week for the first 16 weeks and biweekly for the remaining 8 weeks. The web-based group received weekly PN emails and had access to a general website about dietary change. The control group also received access to the general website. At the end of the trial, the face-to-face group had significantly increased their modified healthy index score (2.2%) compared to PN web-based (−0.18%) (*P* = 0.04) and control groups (−0.54%) (*P* = 0.02) (25).

## Discussion

The interventions reviewed were difficult to compare as they varied considerably in sample size, duration, study design, and contact with participants. Results of the face-to-face nutrition intervention trials indicated that frequent face-to-face nutrition counseling was effective at achieving and maintaining dietary change in both PN and group face-to-face sessions. These results are in line with a recent systematic review of 26 RCT by Mitchell et al. that assessed the effectiveness of PN dietary consultations in primary health care, out of which 18 trials demonstrated significant improvements in either anthropometric outcomes (including weight change) or dietary change including increased fiber, calcium, improvements in salt, and reduced fat intakes compared to comparator groups (39). However, a number of limitations were found in the reviewed trials which included small sample size for long-term trials (25, 36), low numbers of participants in intervention groups which may have underpowered study outcomes (36), control groups receiving no intervention during trial period (21, 26), low adherence rates and high attrition rates (28).

When it came to the delivery of web-based dietary advice, results of the reviewed trials have indicated that PN web-based dietary advice is more effective at improving dietary change, especially consumption of F&V compared to generalized controls. This finding was supported further in a systematic review and meta-analysis of 13 RCT by Celis-Morales et al. that assessed the effectiveness of web-based dietary interventions at enhancing F&V intakes. Results of the systematic review have suggested that personalized web-based nutrition interventions were more effective at improving F&V intakes compared to non-personalized interventions (40).

A number of limitations of the present review should be acknowledged. Firstly, there were differences in the trial designs and a few studies lacked a description of what the control group received during the intervention; this highlights the importance of detailed trial reporting. Moreover, during the review process, it was difficult to determine whether some of the trials were conducted over the web (using a computer) or delivered on a computer-based application as there were no clear definitions used. The lack of detailed descriptions of the type of face-to-face dietary counseling provided e.g., consultation with a health practitioner was a limitation as well as minimal information about the type of usual care provided to control group participants in several trials. It was also difficult to compare the overall effect between the trials as they differed in design. In addition, the majority of the trials have used FFQs to collect dietary data which are subject to recall report bias as they require participants to report dietary intakes over previous weeks or months. A further limitation is the lack of weight-loss data in most of the trials which would have added further data on the effects of dietary change on health outcomes. Moreover, the male:female ratio was not equal as most studies were carried out in women, calling for future trials to target men. As all of the included trials were diet related and based on self-report, this may question the validity of the dietary intake information provided and outcome measures.

Moreover, limited work has focused on comparing the delivery of PN face-to-face and PN web-based dietary interventions, and more comparative trials are needed to demonstrate the strengths and weaknesses of each strategy. Face-to-face trials that targeted dietary change in specific population groups were successful at achieving dietary change; however, face-to-face consultation is costly and is not generally available to the public (20, 41, 42).

## Conclusion

Findings from web-based nutrition interventions and their impact on dietary change suggest that personalized/enhanced web-based nutrition interventions may be successful at inducing short-term dietary change compared to non-personalized dietary interventions. Although face-to-face nutrition interventions were generally successful at enhancing dietary change, those targeting dietary fat yielded inconsistent results which may indicate the need for further long-term research. There still remains insufficient evidence to suggest that web-based PN interventions are as effective as PN face-to-face interventions, therefore, further controlled comparative studies and cost-benefit analysis are needed.

## Author Contributions

BA-A conducted the review of the literature and wrote the manuscript. RF and JL contributed to the drafting of the manuscript. All authors made a critical review of the draft.

## Conflict of Interest

The authors declare that the research was conducted in the absence of any commercial or financial relationships that could be construed as a potential conflict of interest.
